# Decreasing HIV transmissions to African American women through interventions for men living with HIV post-incarceration: An agent-based modeling study

**DOI:** 10.1371/journal.pone.0219361

**Published:** 2019-07-15

**Authors:** Joëlla W. Adams, Mark N. Lurie, Maximilian R. F. King, Kathleen A. Brady, Sandro Galea, Samuel R. Friedman, Maria R. Khan, Brandon D. L. Marshall

**Affiliations:** 1 Brown University School of Public Health, Providence, Rhode Island, United States of America; 2 AIDS Activities Coordinating Office, Philadelphia Department of Public Health, Philadelphia, Pennsylvania, United States of America; 3 Boston University School of Public Health, Boston, Massachusetts, United States of America; 4 National Development and Research Institutes, New York City, New York, United States of America; 5 Division of Comparative Effectiveness and Decision Science, Department of Population Health, New York University, New York City, New York, United States of America; Centers for Disease Control and Prevention, UNITED STATES

## Abstract

**Background:**

Incarceration and HIV disproportionately impact African American communities. The mass incarceration of African American men is hypothesized to increase HIV acquisition risk for African American women. Interventions optimizing HIV care engagement and minimizing sexual risk behaviors for men living with HIV post-incarceration may decrease HIV incidence.

**Methods:**

Using an agent-based model, we simulated a sexual and injection drug using network representing the African American population of Philadelphia. We compared intervention strategies for men living with HIV post-incarceration by the number of averted HIV transmissions to women within the community. Three interventions were evaluated: a 90-90-90 scenario scaling up HIV testing, ART provision, and ART adherence; a behavioral intervention decreasing sexual risk behaviors; and a combination intervention involving both.

**Results:**

The *status quo* scenario projected 2,836 HIV transmissions to women over twenty years. HIV transmissions to women decreased by 29% with the 90-90-90 intervention, 23% with the behavioral intervention, and 37% with both. The number of men living with HIV receiving the intervention needed in order to prevent a single HIV transmission ranged between 6 and 10.

**Conclusion:**

Interventions to improve care engagement and decrease sexual risk behaviors post-incarceration for men living with HIV have the potential to decrease HIV incidence within African American heterosexual networks.

## Introduction

African American communities are disproportionately impacted by HIV and incarceration. Only 12% of the US population identify as African American, yet 44% of individuals with a new diagnosis of HIV and 37% of incarcerated individuals are members of this group [1, 2]. The mass incarceration of African American men is hypothesized to increase HIV acquisition risk for African American heterosexual women; therefore, strategies to reduce HIV risk behaviors related to mass incarceration are needed [3, 4].

Each year, approximately one in five African American men living with HIV within the United States has contact with the criminal justice system [5]. Incarceration frequently complicates HIV care engagement [6–11]. A systematic review summarizing national data on the HIV care cascade before, during, and after incarceration estimated that only 51% of prisoners received HAART during incarceration and only 40% achieved viral suppression [9]. Moreover, any HIV care engagement achieved during incarceration is typically not maintained post-release [7–10]. Iroh et al. estimated that nationwide, only 29% of individuals living with HIV were on HAART and 21% were virally suppressed after release from a correctional facility, compared to general population estimates of 36% and 28%, respectively [9]. Data from this systematic review were not disaggregated by race or gender; however, studies have reported similar or worse outcomes for African American men living with HIV [7, 12]. A growing body of evidence has shown that incarceration can, and often does, lead to disengagement from HIV care and viral rebound for men and women following their sentence and return to the community [6–9, 11, 13–15]. Post-release disengagement from care can lead to HIV disease progression, increases the likelihood of morbidity and mortality for the formerly incarcerated person, and increases the likelihood of HIV transmission to others in the community [9]. Therefore, interventions which optimize HIV care engagement and minimize HIV risk behaviors post-release have the potential to decrease community HIV incidence and HIV-related morbidity.

Modifiable factors can improve HIV care engagement post-release, including the availability of substance use treatment and case management services [7, 9, 16, 17]. Interventions include discharge planning, medical and social support programs, and adherence counseling [16]. Many of the interventions also have the potential to decrease HIV risk behaviors. For example, counseling and connection to services can decrease the likelihood of sharing of injection equipment or condomless sex post-release [18, 19]. Interventions can reduce the risk of HIV transmission to others through achievement of an undetectable viral load and decreasing HIV risk behaviors. Using an agent-based model, this analysis evaluates the ability of post-release intervention strategies for men living with HIV to decrease HIV transmissions to women within the community.

## Methods

### Study design

We used the Treatment of Infection and Transmission in Agent-Based Networks (TITAN) model. TITAN was developed to simulate HIV transmission dynamics within a mature epidemic setting and has been used to evaluate transmission dynamics in various settings [20–22]. As described in a previous study, this agent-based model simulates HIV transmission, disease progression, testing, and treatment, as well as the incarceration of male agents within a virtual population [23]. Based on the 2010 U.S. Census and HIV surveillance data, the model simulated the total population of heterosexual African American women and African American heterosexual or men who have sex with men and women (MSMW) over the age of 18 (n = 457,751) living in Philadelphia. The model was successfully calibrated to HIV prevalence and incidence data for African American women from 2011–2015 using HIV surveillance statistics from the AIDS Activities Coordinating Office within the Philadelphia Department of Public Health. We then used the calibrated model to project twenty years into the future (i.e., 2015–2035) to establish a projected *status quo* scenario and compare hypothetical intervention strategies.

### Model parameters

Behavioral parameters and other elements of agent interactions are summarized in **Table 1**. Parameterization, behavioral processes, and sources are fully described within the Supporting Information (S1–S9 Tables). We modeled a period of high risk lasting approximately six months for both male and female agents as a consequence of either incarceration (for men) or partner incarceration (for women). High-risk male agents had more sexual partners, double the likelihood of acquiring HIV (to approximate the impact of a sexually transmitted infection), and decreased HIV care engagement (if HIV-diagnosed) for six months after release from a correctional facility. Female agents entered a high-risk period upon a partner’s incarceration or the dissolution of a sexual partnership due to incarceration. During this high-risk period, the female agent had an increased number of sexual partners and double the likelihood of acquiring HIV. In addition, we implemented sexual assortative mixing (or the increased likelihood of partnership between two individuals sharing a particular characteristic) by contact with the criminal justice system [24, 25]. Within the model, women were not eligible to experience incarceration. Therefore, contact with the criminal justice system meant experiencing incarceration (men) or having an incarcerated partner (women). For the main analysis, the level of assortative mixing was set to 0.30. As a result, a female agent who previously had an incarcerated partner and is eligible to initiate a new sexual relationship had a 30% probability of pairing with a male agent with a history of incarceration and vice versa. Otherwise, partner selection was purely random. We did not implement assortative mixing for the selection of injecting partners. Assortative mixing related to incarceration and partner incarceration resulted in the creation of higher-risk sexual networks. Increased rates of partner concurrency and relationship turnover emerged from the model rather than resulted from programmed parameters. We did not explicitly model the selection of partners based on perceived HIV-status; however, HIV-diagnosed agents were 50% less likely to engage in unprotected sex with their partners based on prior literature [26].

**Table 1:** Overview of parameters and processes for a model of heterosexual HIV transmission among African American men and women in Philadelphia, PA.

Processes	Description
***Demography***	
Gender	Seeded at baseline based on gender distribution reported in the 2010 U.S. Census.
Mortality	Implemented based on HIV disease stage, use of antiretroviral therapy, and injection drug use.
***Sexual Behavior and Sexual Network***	
Sexual partner preference	Female agents could only partner with male agents. Male agents could partner with female agents or both female and male agents. Seeded stochastically at baseline based on empirical studies.
Condom use	Probability of condom use was based on relationship duration (< or ≥ 1 month), HIV diagnosis, and injection drug use status.
Partner acquisition rate	Agents assigned a personal annual mean number of partners, which was allowed to vary stochastically year-to-year.
Sex frequency	Agents stochastically assigned a desired number of sex acts per partner per year. At the partnership level the resulting number of sex acts represents a compromise between the two partners.
Assortative mixing	Agents searched for partners assortatively based on contact with the criminal justice system. Female agents who experienced partner incarceration had a higher probability of finding a future partner with a history of incarceration and vice versa.
Relationship length	Varies stochastically based on empirical data on mean and median relationship lengths. Agents had a 50% likelihood of relationship dissolution during incarceration.
***Injection Drug Use***	
Rate of injection drug use	Implemented at model initiation based on prevalence of active injection drug use in Philadelphia (173 per 10,000) from 2015 surveillance data.
Frequency of receptive needle sharing	PWID agents engaged in receptive needle sharing stochastically throughout the year with differing probabilities based on gender.
Partner acquisition rate	PWID agents engaged in receptive needle sharing stochastically throughout the year with differing probabilities based on gender.
***Incarceration***	
Incarceration rate	Derived from 2005 data from the Philadelphia Commission on Sentencing for African American men, held constant through model run. Varied by type of correctional facility (jail vs. prison), recidivism status (prior offense vs. first offense). Higher rates for HIV-infected and current PWID male agents.
Sentence length	Derived from 2005 data from the Philadelphia Commission on Sentencing for African American men, held constant through model run. Varied by type of correctional facility (jail vs. prison).
***HIV/AIDS***	
Initial HIV prevalence	Based on 2012 HIV surveillance data for African American men and women in Philadelphia. Higher rates for PWID, MSMW, and male agents who experience incarceration.
Testing	Agents tested stochastically throughout the year with differing probabilities based on gender and injection drug use. Diagnosed agents were less likely to transmit to HIV-negative partners.
HAART	Only HIV-diagnosed agents were eligible to take HAART. Agents on HAART were less likely to transmit to HIV-negative partners.
HAART discontinuation	Only HIV-diagnosed agents receiving HAART were eligible to discontinue. Annual probability of discontinuation differed by gender.
Transmissibility	Based on diagnosis status, HAART adherence, disease stage. Individuals with a current STI are more likely to acquire HIV.

Shaded parameters are those impacted by incarceration or partner incarceration in order to simulate a “high-risk” period.

Abbreviations: HAART- highly active antiretroviral therapy, PWID- persons who inject drugs, MSMW- men who have sex with men and women, STI- sexually transmitted infection

### Model scenarios

As a comparator, we developed a *status quo* scenario to project HIV transmission dynamics over a twenty-year period (2015–2035). For the *status quo* scenario, 69% of all entering non-diagnosed inmates were tested for HIV [27]. Men living with HIV had a probability of initiating HAART (for men newly diagnosed at entry) such that overall 40% were on HAART within the correctional environment at model initialization [9]. In 2015, surveillance reports estimated that 90% of people living with HIV in Philadelphia had received a diagnosis and over 50% were virally suppressed. Therefore, even in the *status quo* scenario, over 90% of HIV-infected agents had been diagnosed and 75% were on HAART while incarcerated. Differences in HIV care between the *status quo* and intervention scenarios were driven by the post-release period. Men already on HAART and those newly diagnosed at entry could discontinue therapy upon release. Specifically, the probability of discontinuing HAART after release was 47.5% by six months post-incarceration [9].

For this analysis, we projected how HIV transmissions to women within the community would be affected by the following scenarios:

1. **90-90-90 Scenario:** Simulated scaling up of HIV testing upon entry (90%), in addition to the scale-up of ART coverage (90%), and the proportion of individuals on ART achieving optimal adherence (90%) by one year post-release for prisoners living with HIV with at least 12-week sentences. Treatment commenced immediately upon entry into a correctional facility and continued through a minimum of one year post-release. After one year, the rate of discontinuation was set equal to that for men without a history of incarceration within the community. Scaling up of HIV testing and ART coverage began at model initialization (i.e., 2011) in order to accomplish the ‘90-90-90’ targets by 2015.
2. **Behavioral Intervention:** Simulated the provision of a hypothetical HIV behavioral intervention that prevented an increase in post-release HIV risk behaviors (i.e., number of sex partners and assortative mixing) for prisoners living with HIV with a sentence of at least 12 weeks. Sexual risk behaviors for agents receiving the intervention were equivalent to that of the agent before incarceration. The probability of HIV care engagement did not differ from that of the *status quo* scenario. Intervention began at the start of the year 2015.
3. **Combination Intervention:** Simulated the provision of a strategy combining the 90-90-90 and the behavioral interventions.

Analyses based on the above three scenarios were run 200 times with ¼ population size (n = 110,000). This research was conducted using computational resources and services at the Center for Computation and Visualization, Brown University. As an epidemic modeling study, the study did not require review from an institutional review board.

### Sensitivity analyses

Due to uncertainty regarding the causal effect of incarceration on post-release risk behavior, we performed sensitivity analyses changing the duration of male high-risk behavior post-release, and additional sensitivity analyses in which the number of sexual partners was not increased post-release. In addition, we performed sensitivity analyses varying the level of assortative mixing (0, 0.50) and with a shorter model run. In order to evaluate how network metrics were impacted by this parameter, we visually inspected samples of networks produced by varying levels of assortative mixing within the *status quo* scenario.

### Outcomes

In order to describe the potential effect of the modeled interventions on HIV acquisition in African American women, we compared the *status quo* scenario to the three counterfactual scenarios. For each scenario, we projected the mean number of HIV transmissions to African American women and women with a history of high-risk behavior due to partner incarceration over the 20-year period. Model scenarios were compared by calculating the average number of averted infections and the number of intervention participants needed in order to prevent one HIV transmission. As done in previous modeling studies to account for the stochastic framework of these models, we present means with 95% simulation intervals (SI), which reflect the 95% upper and lower limits of the simulated output [28].

## Results

We used HIV surveillance statistics for Philadelphia to project trends in HIV transmission dynamics among African Americans for a twenty year study period (i.e., 2015–2035). Over the study period, there were 2,836 (95% Simulation Interval [SI]: 2,539–3,135) projected HIV transmissions or an average incidence rate of 54 per 100,000 women for the female African American population. Over twenty years, 22% of men and 62% of men living with HIV experienced at least one period of incarceration. In total, 8% (n = 21,589) of women experienced a period of high-risk behavior due to a partner’s incarceration, and of those, 470 (95% SI: 376–573) acquired HIV. There were 479 (95% SI: 382–580) HIV transmissions to men who experienced incarceration over the study period.

**Fig 1:**
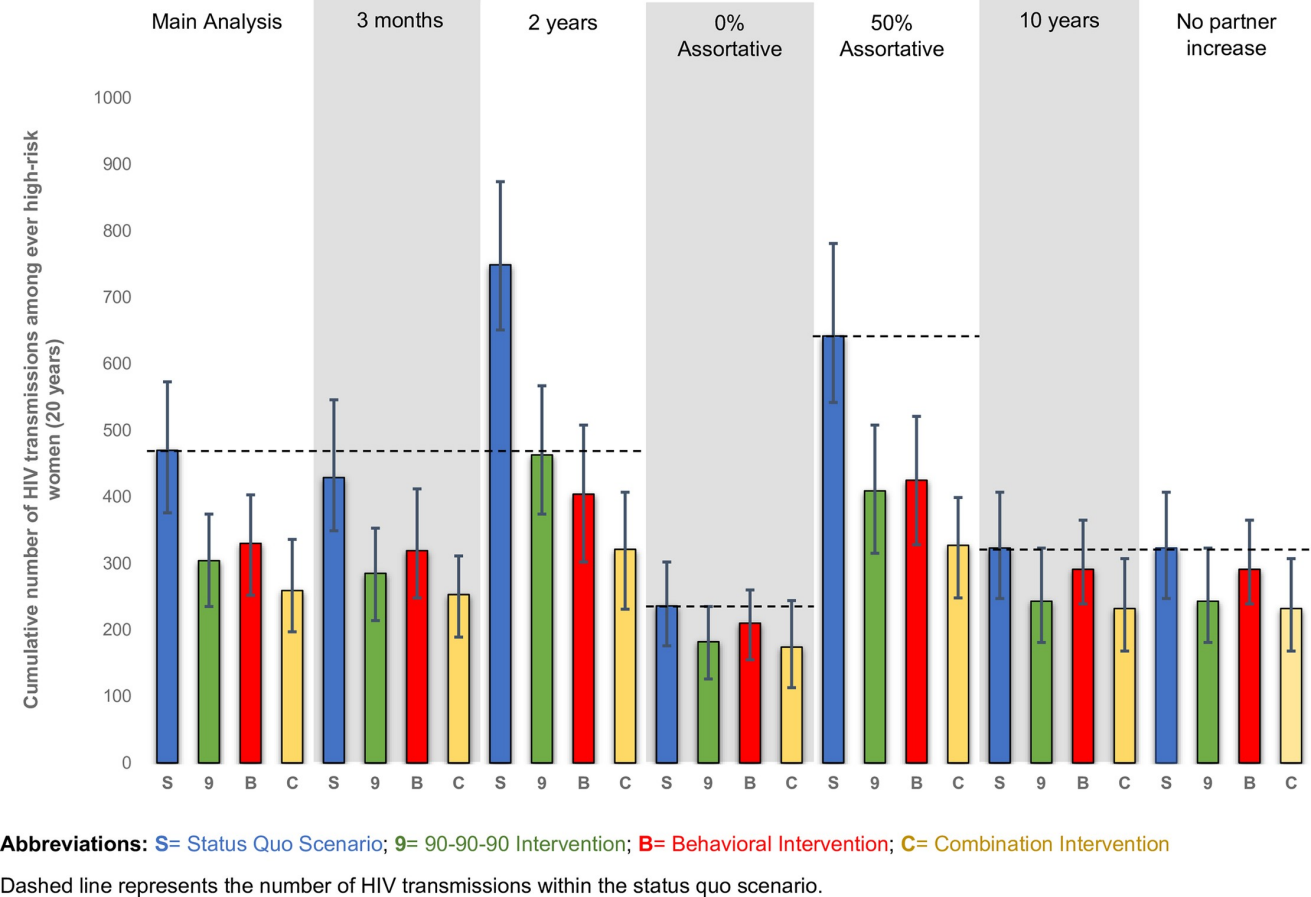
Cumulative number of HIV transmissions among African American women who experienced a partner’s incarceration.

**Fig 2:**
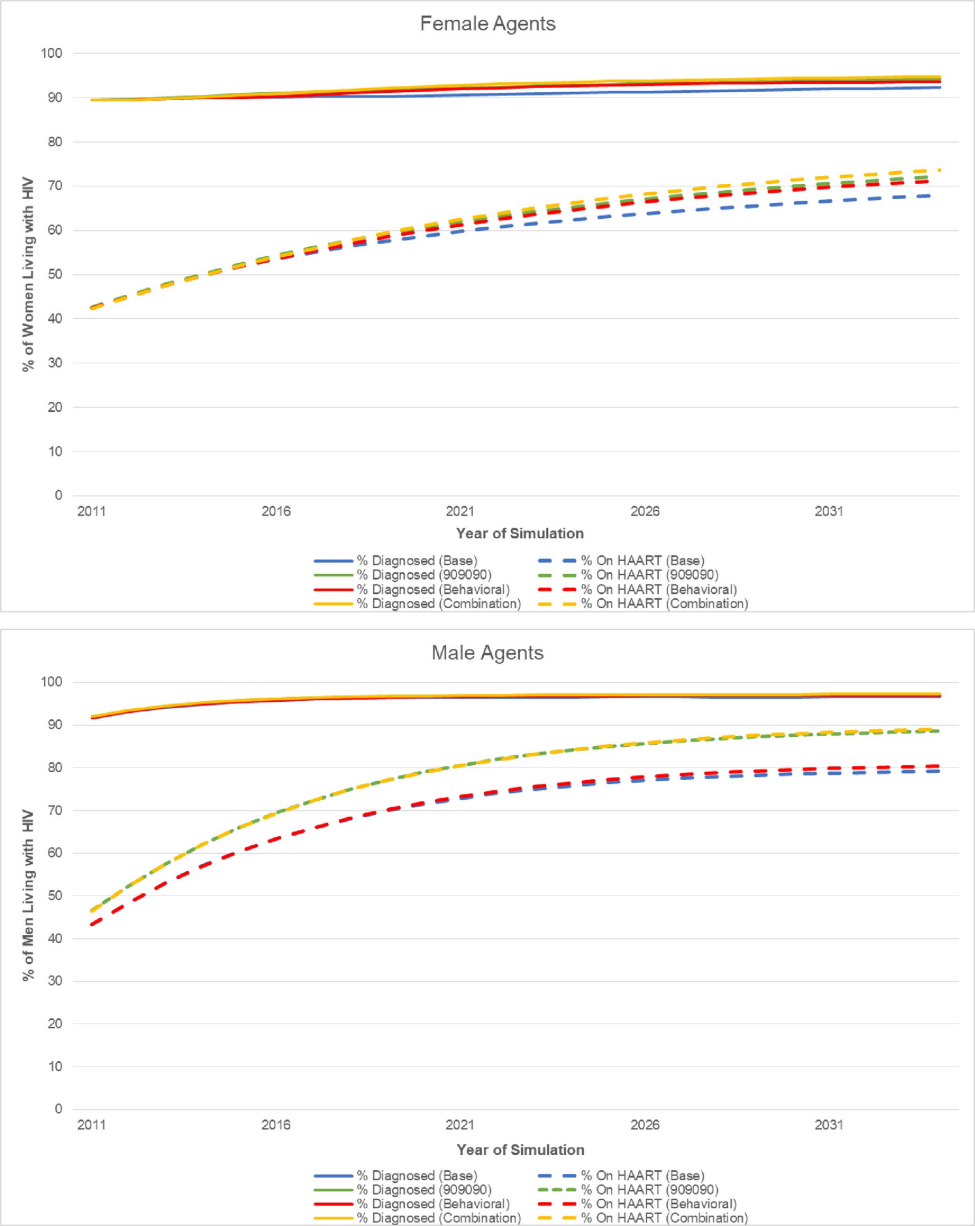
Proportion of people living with HIV who are diagnosed and on HAART by scenario.

The 90-90-90 scenario resulted in 2,011 (95% SI: 1,754–2,281) HIV transmissions to women over the study period or 825 (29%) averted HIV transmissions. There were 304 transmissions to women who experienced a partner’s incarceration. Over the twenty year study period, 6,500 men living with HIV received the hypothetical intervention, leading to an average number needed to treat (NNT) to avert one infection of 8 (calculated as the number of men receiving intervention [n = 6,500] divided by the number of averted HIV transmissions among women [n = 825]). The behavioral scenario resulted in 2,174 (95% SI: 1,898–2,432) HIV transmissions to women or 662 (23%) averted transmissions and 330 transmissions to women who experienced partner incarceration. The NNT was 10. The combination intervention proved the most efficacious with 1,784 (95% SI: 1,560–2,045) HIV transmissions to women or 1,052 (37%) averted HIV transmissions, 259 transmissions to women who experienced partner incarceration, and a NNT of 6. Results for women overall are summarized in **Table 2.** In **Fig 1,** intervention scenarios are compared for the subset of women who experienced a high-risk period due to a partner’s incarceration. Decreases in HIV transmission were partly driven by the increased HIV care engagement of male agents. As shown in **Fig 2**, both the 90-90-90 and combination scenarios increased the proportion of HIV-infected male agents on HAART compared to the *status quo* and behavioral intervention scenarios.

**Table 2:** Average number of cumulative new HIV infections and mean number of averted HIV infections among African American women over 20-year period by scenario.

	S*tatus quo* scenario (N, 95% Simulation Interval [SI])	90-90-90 intervention (N, 95% SI)	Infections averted (N, %)	HIV risk behavior intervention (N, 95% SI)	Infections averted (N, %)	Combination intervention (N, 95% SI)	Infections averted (N, %)
**Main Analysis**							
*Duration of male high risk behavior set to six months*, *assortative mixing set to 30%*	2,836 (2,539–3,135)	2,011 (1,754–2,281)	825 (29%)	2,174 (1,898–2,432)^a^	662 (23%)^a^	1,784 (1,560–2,045)	1,052 (37%)
**Sensitivity Analyses:**							
*Duration of male high risk behavior*							
Three months	2,667 (2,390–2,974)	1,921 (1,672–2,142)	746 (28%)	2,144 (1,831–2,444)	523 (20%)	1,774 (1,537–2,050)	893 (33%)
Two years ^b^	3,802 (3,490–4,171)	2,561 (2,281–2,881)	1,241 (33%)	2,484 (2,050–2,650)	1,318 (35%)	2,095 (1,886–2,415)	1,707 (45%)
*Assortative mixing*							
0%	2,390 (2,138–2,696)	1,844 (1,646–2,054)	546 (23%)	2,073 (1,798–2,331)	317 (13%)	1,735 (1,457–1,974)	655 (27%)
50%^b^	3,228 (2,915–3,587)	2,207 (1,898–2,549)	1,021 (32%)	2,310 (2,050–2,650)	918 (28%)	1,886 (1,634–2,129)	1,342 (42%)
*Length of model run*							
10 years	1,165 (1,040–1,336)	891 (748–1,071)	274 (24%)	940 (819–1,092)	225 (19%)	805 (664–945)	360 (31%)
*No increase in sexual partners during high-risk period*	2,810 (2,528–3,129)	2,219 (1,961–2,499)	591 (21%)	2,452 (2,192–2,767)	358 (13%)	2,114 (1,835–2,415)	696 (25%)

^a^ Due to computing time limitations, the number of completed runs was 195 (rather than 200).

^b^ Due to computing time limitations, the number of completed runs was less than 100. For the two year male duration of high risk behavior sensitivities, the number of completed runs was 95 for the 90-90-90 and 97 for the behavioral scenarios. For the 50% assortative mixing sensitivities, the number of completed runs was 98 runs for the status quo, 96 for the 90-90-90, 98 for the behavioral, and 97 for the combination intervention.

**Fig 3:**
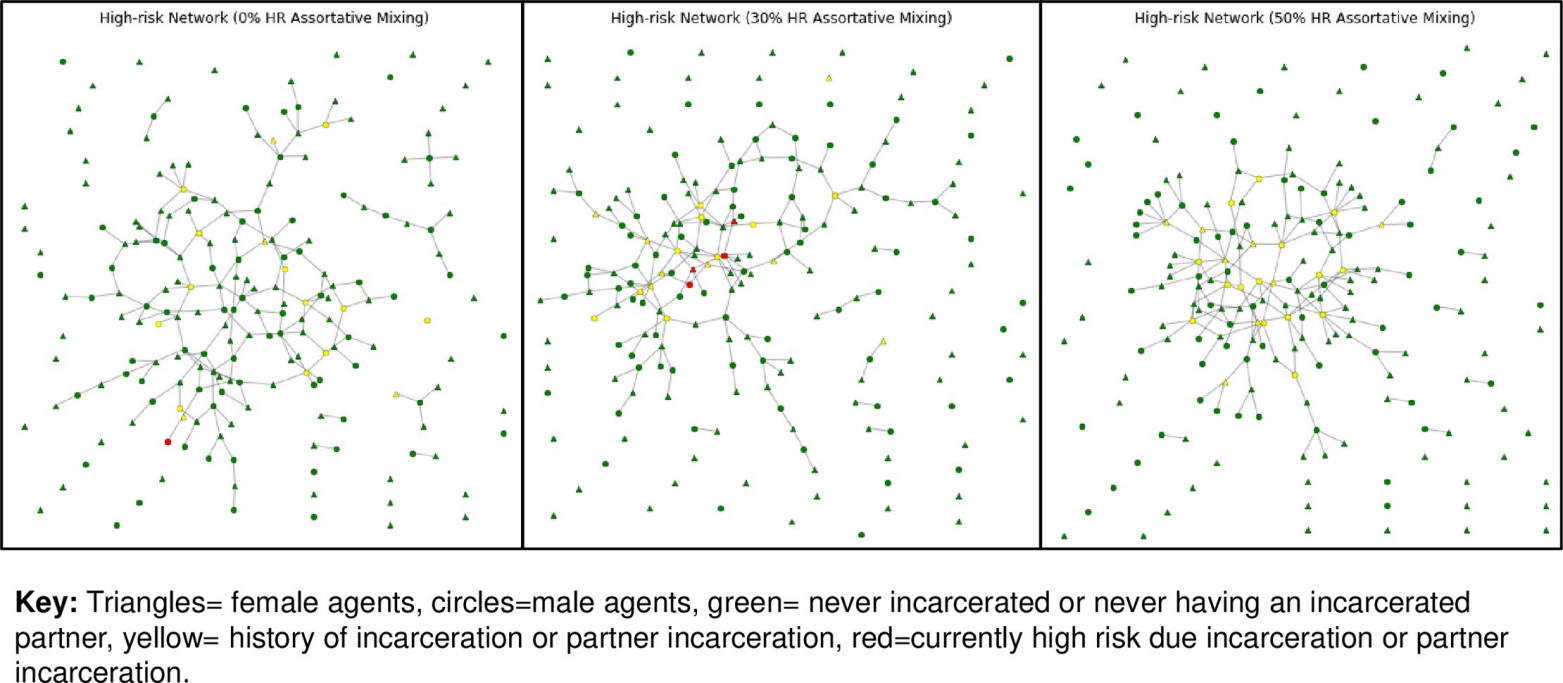
Network sample with 200 agents at year 10 at varying levels of assortative mixing.

Sensitivity analyses demonstrated that intervention effects were attenuated with a shorter duration of male high-risk behavior post-incarceration, at 0% assortative mixing (or purely random mixing), and without the increase in sexual partners during the high-risk period. An increase in HIV transmissions to women as well as increased intervention efficacy occurred in the analyses with longer duration of male high-risk behavior and at higher rates of assortative mixing. **Fig 3** presents visualizations of the networks produced by increasing levels of assortative mixing within the *status quo* scenario. As assortative mixing increased, agents who had ever been incarcerated or experienced partner incarceration (indicated in yellow) became more densely connected and confined to one large component. A consequence of this is that interventions focused on these high-risk networks have greater potential to prevent transmission.

## Discussion

These findings emphasize the potential role that prison- and community-based interventions for men living with HIV post-release have in decreasing HIV transmissions within African American heterosexual networks. Both the 90-90-90 and behavioral interventions decreased the total number of HIV transmissions to women within the community by over 20% while an intervention strategy combining them both decreased the number of transmissions by nearly 40%.

Implementing a 90-90-90 scenario resulted in a significant decrease in the number of HIV transmissions. Achieving a 90-90-90 scenario requires universal HIV testing within the correctional facility and nearly ideal HIV care engagement post-release. Existing studies suggest that achieving a high level of HIV testing is feasible. Adoption of opt-out HIV testing resulted in over 95% of inmates completing testing in Rhode Island jails and within the North Carolina prison system [29, 30]. In addition, the acceptability of HIV testing is likely to be high. A study in North Carolina found that 89% of prisoners wanted to be tested for HIV [31]. In contrast, optimizing HIV care engagement post-release poses significant challenges. Interventions have utilized enhanced case management, patient navigation, discharge planning services, and a combination thereof with mixed success [9, 12, 17, 32–34]. The percentage of individuals retained in care post-release within these interventions ranged from 28–96% [9]. A recent study by Wohl *et al*. reported 60% of study participants maintained viral suppression at six months post-incarceration after receiving motivational interviewing, a needs assessment, and medical care coordination [35]. However, this was not an improvement from the control arm (61% suppressed) [15]. Another intervention involving transitional care coordination in New York reported 93% of individuals seen at six months post-incarceration were taking ART [36]. However, the percentage with an undetectable viral load was lower (35%) and there was significant loss to follow-up. Additional work is needed to clarify what intervention components lead to high levels of retention and viral suppression. Advances in HIV tools such as the introduction of long-acting injectable antiretrovirals might facilitate the achievement of viral suppression rates 90% and higher [37]. In summary, achieving a high level of HIV care engagement post-release will likely require a comprehensive and flexible set of interventions.

Implementing a hypothetical behavioral intervention for men living with HIV post-release led to a smaller but substantial decrease in HIV transmission. While the majority of interventions focus on improving HIV care engagement, counseling on risk behaviors is often a component [12, 18]. Grinstead et al. evaluated an pre-release intervention program for men living with HIV from 1996–1998 [38]. In comparison to the control group, intervention participants reported greater use of community resources (e.g., financial aid, counseling) and reduced post-release sexual and drug-related risk behavior [38]. Intervention participants were more likely to have used a condom the first time they had sex post-release and less likely to have first had sex with someone they just met or a sex worker [38]. A more recent randomized control study by Reznick et al. compared an individually-based behavioral intervention to an ecosystem-based intervention and found reduced sexual risk behaviors in both intervention arms [18]. Assuming accurate self-report, these findings suggest that decreases in sexual risk behavior in this patient population are feasible.

Within our model, a combination intervention strategy resulted in the greatest reduction in HIV transmissions. In total, 37% of the HIV transmissions to women predicted in the *status quo* scenario were averted. Efforts to improve HIV care engagement and decrease HIV risk behaviors can be synergistic [18]. Our results suggest that interventions combining care engagement and behavioral components are likely to optimize decreases in HIV transmissions. While not the focus on the present analysis, these interventions would also benefit the men receiving the intervention by reducing the morbidity and mortality related to disengaging from HIV care.

All of the intervention scenarios were effective at both shorter and longer durations of male high-risk behavior post-incarceration. Lengthening the duration of male high-risk behavior increased the number of HIV transmissions to women as well as the intervention effectiveness. Similarly, highly assortative mixing increased the overall number of HIV transmissions and intervention effectiveness. Since men living with HIV were more likely to be incarcerated and HIV risk behaviors (i.e., injection drug use, partner concurrency) were more prevalent among persons with a history of incarceration, assortative mixing by incarceration and partner incarceration resulted in the creation of high risk networks. These networks, in turn, drove HIV transmission. The role of sexual mixing by individual characteristics in driving the overall incidence of HIV is well established [39, 40]. Sexual partner selection can be assortative by sociodemographic factors including income level and illicit drug use [25]. Since our agent population is racially homogenous, 100% assortative mixing based on race is implemented by default. Our findings demonstrate how HIV incidence is impacted by assortative mixing related to contact with the criminal justice system when incarceration is a common experience. At a moderate level of assortative mixing (0.3), HIV rates were increased compared to the network with purely random partner selection. This finding may be a function of the relatively low prevalence and incidence of HIV within our agent population as other studies have found lower rates of transmission as assortative mixing increases in populations with a higher disease incidence [40, 41]. Interventions had a greater impact at higher levels of assortative mixing, particularly for women who had an incarcerated partner. Therefore, the existence of an intervention effect may depend on the interconnectedness or existence of higher risk networks related to incarceration. These results relate to a 2015 study using an agent-based model which found that incarceration could increase community-level HIV risk by changing sexual partnership patterns [42]. However, additional research is needed to better understand how incarceration influences sexual networks and post-release risk behavior in the real world.

Our study is subject to several limitations. Our simulations projected twenty years into the future assuming that key parameters, including discontinuation of HAART within the community and trends in HIV incidence remained constant. Due to the long time frame, the reported HIV transmissions and the number of incarcerations within the *status quo* scenario are unlikely to reflect real-world conditions at the end of 2035. However, these projected statistics are useful in comparing differing intervention strategies to one comparator scenario. We also performed a sensitivity analysis using a short timeframe (10 years), which resulted in similar findings with slightly attenuated intervention effects. An additional limitation relates to model calibration. In order to recreate trends in HIV prevalence and incidence seen among African American women in Philadelphia, the simulated incidence and prevalence levels for men were lower than that reported. However, these surveillance-reported incidence and prevalence rates included men who exclusively have sex with other men and overestimates HIV trends for heterosexual and MSMW men. Lastly, we assumed that sexual contact and the sharing of injection materials ceased during incarceration despite evidence to the contrary [43–45]. We were not able to model these behaviors due to lack of empirical data. However, studies have shown the majority of HIV transmission occurs outside of correctional facilities [46, 47].

One practical limitation is that this analysis assumes the capacity for correctional facilities or community-based organizations to provide services for all inmates with a 12-week or longer sentence. This assumption may not be reasonable for certain settings as the provision of services is dependent on the availability of funding and other factors. Intervention strategies may require adaptation to resource-limited settings in order to successfully link inmates to HIV care post-release. A related challenge is the ability for interventions to achieve the level of care engagement (i.e., 90%) at twelve months post-release modeled within the analysis. While smaller intervention studies have reported success achieving levels of HAART coverage over 90%, most studies report lower levels of coverage and viral suppression [9]. Alternative strategies may be needed in order to retain individuals in HIV care post-release. Nonetheless, we sought to model a scenario that, although optimistic, is in line with UNAIDS targets and has been reported in some studies. Future work is needed to determine whether the 90-90-90 goals are feasible and achievable in the diverse types of correctional settings across the United States.

Finally, like all simulations, this agent-based model is not able to fully capture all factors related to incarceration or partner incarceration and relies on certain assumptions. The use of an ABM required defining the causal effect of incarceration and partner incarceration on sexual risk behaviors. The use of observational studies to parameterize these causal processes may result in bias, particularly when the studies were derived from populations in other settings [48]. For example, we based the number of sexual partners for high-risk female agents on a small qualitative study based in Atlanta [49]. We have tried to mitigate this bias by fully describing the source of parameters within the supplementary materials and performing sensitivity analyses. In a previous study using this model, we ran a series of sensitivity analyses to understand what factors related to incarceration drove HIV transmission to women and how HIV transmission dynamics would vary in response to uncertainty related to these factors [23]. The duration of male high-risk sexual behavior and HIV care engagement post-release as well as STI infection had the largest impact on HIV transmission to women and informed sensitivity analyses included in the present analysis. We also performed sensitivity analyses varying assortative mixing as this variable captures some of the unknown potential confounders related to incarceration that could drive HIV transmission (for example, living in high-poverty neighborhoods, shared experiences of stigma and discrimination). We also note that riskier post-release sexual behaviors may in fact be caused by another factor (e.g., substance use, exchange sex, etc.) preceding incarceration or that these behaviors may be highly correlated with, but not caused by, incarceration. Nonetheless, the results of our sensitivity analyses show that, while the intervention effects are attenuated when no increase in post-release risk behavior is assumed, programs for HIV-infected men post-release might avert a significant number of new infections among women. Additionally, relationship duration may have been underestimated and partnership acquisition rates overestimated due to the use of empirical data from studies of higher risk individuals. This may have resulted in overestimating the overall number of HIV transmissions within the study population and/or within subsets of the population during the study period. Another limitation relates to determinants of our outcome that are not accounted for within the model. For example, commercial sex and non-injection substance use were not explicitly modeled. Both commercial sex [50] and non-injection substance use [51] are associated with an increased risk of HIV infection and incarceration. As a consequence, the simulated effect of incarceration on HIV transmission may be conservative. Sentencing information used to parameterize the model were averages shared by the Philadelphia Commission on Sentencing and did not account for time spent within correctional facilities awaiting trial or sentencing or individuals who were released on parole before completing their sentence. Uncertainty regarding the number of men spending time within correctional facilities could led to a more conservative or more extreme simulated effect.

Despite these limitations, this model offers the opportunity to evaluate potential intervention strategies and assist in resource-allocation decisions. These strategies can complement work to decrease incarceration rates and recidivism by addressing structural factors including racism and poverty. For example, transportation assistance can help individuals attend job interviews as well as medical appointments for HIV care. In this manner, both the probability of HIV transmission and incarceration rates can be decreased at the community-level. Additional work with this model is being done to evaluate the effect of decarceration as well as the potential usefulness of pre-exposure prophylaxis (PrEP) in decreasing transmissions.to women with partners who have a history of incarceration. While MSM were not included within this model, these findings suggest that HIV care engagement and behavioral interventions for MSM living with HIV and experiencing incarceration may reduce HIV transmission to their male partners, and should be a subject of future research. Strengths of this study include the use of HIV surveillance statistics and the inclusion of detailed parameters related to incarceration, including sentence lengths for prison and jail and the probability of recidivism by previous criminal justice involvement, specific to Philadelphia for African American men.

In summary, interventions to improve HIV care engagement and decrease sexual risk behaviors for African American men living with HIV post-release have the potential to significantly decrease HIV transmissions to African American women in the community. Assortative mixing by contact with the criminal justice system modified the effectiveness of these interventions and additional research is needed to understand the characteristics of sexual partners and the resulting networks for persons with criminal justice system involvement.

## Supporting information

S1 Table Fixed and time variant state variables of agents. (PDF)

S2 Table Initial model conditions representing start of year 2011. (PDF)

S3 Table Parameter estimates related to HIV screening and treatment. (PDF)

S4 Table Parameters and data sources for HIV disease progression and mortality. (PDF)

S5 Table Parameters and data sources for HIV transmission. (PDF)

S6 Table Parameter estimates for sexual behavior. (PDF)

S7 Table Parameters related to injection drug use. (PDF)

S8 Table Parameters and data sources for the impact of incarceration or partner incarceration. (PDF)

S9 Table Parameters varied within sensitivity analyses. (PDF)
