# Supplementary material for: Decreasing HIV transmissions to African American women through interventions for men living with HIV post-incarceration: An agent-based modeling study
Source: PLoS One. 2019 Jul 15;14(7):e0219361. doi: 10.1371/journal.pone.0219361 (PMC6629075; doi:10.1371/journal.pone.0219361)
Supplement: S1 Table — (PDF) [file pone.0219361.s001.pdf]

S1 Table. Fixed and time variant state variables of agents.

State Variable	Fixed	Time Variant	Data Source
Sex	X		2000 U.S. Census
Sexual Partner Type	X		Lieb *et al.*[11], Dyer *et al.*[8]
PWID Status		X	AACO
HIV Serostatus		X	AACO
HIV Diagnosis Status		X	Marks *et al.*[12]
HAART Adherence		X	AACO
AIDS Status		X	AACO
Incarceration Status		X	Goldkamp *et al*.[13], PCS[14], Mauer *et al*.[15]

Abbreviations: PWID- person who injects drugs, AACO- AIDS Activities Coordinating Office within the Philadelphia Department of Public Health; HAART- highly active antiretroviral therapy; PCS- Philadelphia Commission on Sentencing
