# Supplementary material for: Decreasing HIV transmissions to African American women through interventions for men living with HIV post-incarceration: An agent-based modeling study
Source: PLoS One. 2019 Jul 15;14(7):e0219361. doi: 10.1371/journal.pone.0219361 (PMC6629075; doi:10.1371/journal.pone.0219361)
Supplement: S2 Table — (PDF) [file pone.0219361.s002.pdf]

S2 Table. Initial model conditions representing start of year 2011.

Variable	Base estimate				Data Source
	Male Agents	*Male PWID[a]*	*Female Agents*	*Female PWID[a]*	
Community size (%)	41.4%	1.73%	58.5%	1.73%	Calculated, U.S. Census 2010, Lieb *et al.*[11], AACO
HIV prevalence (%)	3.7%[b]	15%	1.4%	15%	Calculated, AACO
Proportion of HIV-infected individuals with HIV diagnosis	90%		90%		AACO
Proportion of HIV-diagnosed individuals on HAART (%)	45%		51%		Assumed/calibrated, AACO
AIDS prevalence	67%		57%		Calculated, AACO
Proportion incarcerated (%)	2.74%	Varied [c]	n/a	n/a	Estimated, Goldkamp *et al.*[13]

[*a*] PWID agents are a subset of the gender (male or female) agent class. Parameters are equivalent to that of the male or female agent class unless specifically noted.
[b] HIV surveillance data reported HIV prevalence for African American men including MSM.
[c] PWID agents had an annual probability of being incarcerated of 42.8% based on the Philadelphia NHBS-IDU 2015 survey.[16] This was not a race-specific estimate. The exact starting proportion varied slightly but was based on this probability.
