# Supplementary material for: Decreasing HIV transmissions to African American women through interventions for men living with HIV post-incarceration: An agent-based modeling study
Source: PLoS One. 2019 Jul 15;14(7):e0219361. doi: 10.1371/journal.pone.0219361 (PMC6629075; doi:10.1371/journal.pone.0219361)
Supplement: S3 Table — (PDF) [file pone.0219361.s003.pdf]

S3 Table. Parameter estimates related to HIV screening and treatment.

Variable	Base estimate				Data Source
	Male Agents	*Male PWID[a]*	*Female Agents*	*Female PWID[a]*	
HIV testing (monthly %)	3.43%	5.29%	3.93%	5.29%	NHBS[16,17]
Proportion of HIV-infected agents with HIV diagnosis	90%		90%		AACO
Proportion of HIV-diagnosed agents on HAART	Increases over time				AACO
Discontinuation of HAART (% per year)	42%		52%		Robison *et al.*[18], Adieh-Grant *et al.*[19]

[a] PWID agents are a subset of the gender (male or female) agent class. Parameters are equivalent to that of the male or female agent class unless specifically noted.
