# Supplementary material for: Decreasing HIV transmissions to African American women through interventions for men living with HIV post-incarceration: An agent-based modeling study
Source: PLoS One. 2019 Jul 15;14(7):e0219361. doi: 10.1371/journal.pone.0219361 (PMC6629075; doi:10.1371/journal.pone.0219361)
Supplement: S4 Table — (PDF) [file pone.0219361.s004.pdf]

S4 Table. Parameters and data sources for HIV disease progression and mortality.

Variable	Base Estimate			Source
	Male Agents	*Female Agents*	*PWID Agents*	
**Progression to AIDS (annual probability)[a]**				Egger *et al.*[21]*,* Moss *et al*.[22]*,* Porter *et al.*[23]
Not on ART	0.005			
0% – 29% adherent to ART	0.005			
30% – 49% adherent to ART	0.0039			
50% – 69% adherent to ART	0.0032			
70% – 89% adherent to ART	0.0025			
≥90% adherent to ART	0.0008			
**All-Cause Mortality Rate (per 1,000 person-years)**				
Among HIV negative agents	7.31	3.77	21.7	NCHS[25], Mathers *et al.*[26]
Among HIV positive agents, not on ART	16.5	16.5	65.1	Estimated: Siddiqi *et al*.[27], Mathers *et al.*[26]
Among HIV positive agents, on ART	7.31	3.77	43.4	NCHS[25], Siddiqi *et al*.[27], Lappalainen *et al.*[28]
Among Agents diagnosed with AIDS	33	33	65.1	Estimated: Siddiqi *et al*.[27], Mathers *et al.*[26]

Abbreviations: HIV – human immunodeficiency virus; HAART –highly active antiretroviral therapy, NCHS – National Center for Health Statistics.

[a] HIV surveillance data used to estimate the proportion of agents achieve ≥90% of adherence upon initiating HAART (the remaining proportion are assigned to four other quartiles [0% - 29%, 30% - 49%, 50% - 69%, 70% - 89%] with equal probability)
