# Supplementary material for: Decreasing HIV transmissions to African American women through interventions for men living with HIV post-incarceration: An agent-based modeling study
Source: PLoS One. 2019 Jul 15;14(7):e0219361. doi: 10.1371/journal.pone.0219361 (PMC6629075; doi:10.1371/journal.pone.0219361)
Supplement: S5 Table — (PDF) [file pone.0219361.s005.pdf]

S5 Table. Parameters and data sources for HIV transmission.

Variable	Base Estimate		Source
	Male Agents	*Female Agents*	
HIV transmission risk per unprotected vaginal sex act (chronic phase) by adherence to HAART level			Gray *et al.*[32], Quinn *et al.*[33]
Not on HAART	0.0010		
0-29% adherent	0.0010		
30-49% adherent	0.0008		
50-69% adherent	0.0004		
70-89% adherent	0.0002		
≥90% adherent	0.0001		
HIV transmission risk per needle or works injection sharing act (chronic phase) by adherence to HAART level			Kaplan *et al.*[38], Baggaley *et al.*[39], Hudgens *et al.*[40]
Not on HAART	0.0070		
0-29% adherent	0.0070		
30-49% adherent	0.0056		
50-69% adherent	0.0028		
70-89% adherent	0.0014		
≥90% adherent	0.0002		
Increase in infectivity during acute stage infection	4.3		Bellan *et al.*[35], Wawer *et al.*[34]
Early phase duration (months)	3		Bellan *et al.*[35], Wawer *et al.*[34]
