# Supplementary material for: Decreasing HIV transmissions to African American women through interventions for men living with HIV post-incarceration: An agent-based modeling study
Source: PLoS One. 2019 Jul 15;14(7):e0219361. doi: 10.1371/journal.pone.0219361 (PMC6629075; doi:10.1371/journal.pone.0219361)
Supplement: S6 Table — (PDF) [file pone.0219361.s006.pdf]

**S6 Table**. Parameter estimates for sexual behavior.

| Variable | Base estimate | | | | Data Source |
|---|---|---|---|---|---|
| | *Male Agents* | *Male PWID*[a] | *Female Agents* | *Female PWID*[a] | |
| Sexual partners over one year, median (IQR) | n/a | 2 (1, 4) | n/a | 3 (1, 15) | NHBS (national)[16] |
| Current partners at any one time point, mean (SD) | 0.76 (1.04) | n/a | 0.72 (1.00) | n/a | Calibrated |
| Cumulative new partners over 6 months, median (IQR) | 0.30 (0.15-0.6) | n/a | 0.30 (0.08-0.53) | n/a | Calibrated |
| Relationship duration | 1-6 months (58.5%), 7-12 months (11.6%), 1-2 years (12.1%), 2-3 years (6%), 3-4 years (11.8%) | | | | Calibrated |
| Mean number of vaginal sex acts per month (95% CI) | 4.9 (4.3, 5.5) | | 4.9 (4.3, 5.6) | | Leichliter *et al.*[48] |
| Median number of vaginal sex acts per month (95% CI) | 2.5 (1.9, 3.0) | | 1.9 (1.5, 2.3) | | Leichliter *et al.*[48] |
| Probability of unprotected sex with main partner (relationship duration ≥ 1 month) | 90.5% | 87% | 90.5% | 87% | Sionean *et al.*[42], NHBS (national)[16] |
| Probability of unprotected sex with main partner (relationship duration <1 month) | 72.5% | 74.5% | 72.5% | 74.5% | Sionean *et al.*[42], NHBS (national)[16] |
| Assortative mixing | 0.3 | | 0.3 | | Estimated, Khan *et al.*[47] |

Abbreviations: SD- standard deviation, IQR- interquartile range, CI- confidence interval
