# Supplementary material for: Decreasing HIV transmissions to African American women through interventions for men living with HIV post-incarceration: An agent-based modeling study
Source: PLoS One. 2019 Jul 15;14(7):e0219361. doi: 10.1371/journal.pone.0219361 (PMC6629075; doi:10.1371/journal.pone.0219361)
Supplement: S7 Table — (PDF) [file pone.0219361.s007.pdf]

S7 Table. Parameters related to injection drug use.

Variable	Base estimate		Data Source
	Male PWID Agents	*Female PWID Agents*	
Receptive sharing of syringes, injecting equipment, or using a syringe to divide drugs (annual probability)	69%	63%	NHBS IDU-2015 Philadelphia
Median number of injection partners in past year with receptive syringe sharing (Q1, Q3)	2 (1, 3.5)	3 (1, 3)	NHBS IDU-2015 Philadelphia
Mean number of needle-sharing acts per day	4	4	Johnson *et al*.[51]
Probability of cessation of injection drug use per month	0.2%		Galai *et al*.[50]
