# Supplementary material for: Decreasing HIV transmissions to African American women through interventions for men living with HIV post-incarceration: An agent-based modeling study
Source: PLoS One. 2019 Jul 15;14(7):e0219361. doi: 10.1371/journal.pone.0219361 (PMC6629075; doi:10.1371/journal.pone.0219361)
Supplement: S8 Table — (PDF) [file pone.0219361.s008.pdf]

S8 Table. Parameters and data sources for the impact of incarceration or partner incarceration.

Variable	Base estimate			Data Source
	Male Agents	*PWID Male Agents*	*Female Agents*	
Proportion currently incarceration (%)	2.7-2.8%	See below	n/a	Calculated: Goldkamp *et al.*[13], Mauer *et al.*[15], Sakala *et al.*[54]
Annual probability of incarceration (%)	n/a	42.8%	n/a	NHBS IDU-2015 Philadelphia
HIV prevalence rate ratio for incarcerated vs. non-incarcerated men	5.0		n/a	Maruschak *et al.*[55]
Annual rate of incarceration in jail per 100,000 people for first-time offenders	100		n/a	PCS[14]
Annual rate of incarceration in jail per 100,000 people for those with a prior record	276		n/a	PCS[14]
Average length of minimum jail sentence (months)	8		n/a	PCS[14]
Average length of maximum jail sentence (months)	21.6		n/a	PCS[14]
Annual rate of incarceration in prison per 100,000 for first-time offenders	75		n/a	PCS[14]
Annual rate of incarceration in prison per 100,000 for those with a prior record	251		n/a	PCS[14]
Average length of minimum prison sentence (months)	45.6		n/a	PCS[14]
Average length of maximum jail sentence (months)	96		n/a	PCS[14]
HIV testing in correctional facility (probability upon intake)	69%		n/a	Beckwith *et al.*[56]
Percent of HIV-infected inmates on HAART while incarcerated	40%		n/a	Iroh *et al.*[59]
Percent of main relationships dissolving during incarceration	50%		n/a	Khan *et al.* 2011[57], Khan *et al.* 2018[58]
Current partners at start of high-risk period, mean (SD)	0.84 (1.2)		1.8 (1.5)	Calibrated
Cumulative new partners over 6 months, median (IQR)	5.2 (5.1-5.3)		5.3 (5.1-5.5)	Calibrated
Increase in HIV acquisition risk due to current STI	Doubled		Doubled	Rogers *et al.*[64], van de Wijgert *et al.*[66]

Percent of HIV-infected inmates maintained on HAART at six months post-release	21%	n/a	Iroh *et al.*[59]

Abbreviations: n/a- not applicable; PCS- Philadelphia Commission on Sentencing

[a] All male agents released from prison or jail are high-risk for six months following release. 30% of women with a main partner who is incarcerated are high-risk immediately upon a partner's incarceration and 50% are high-risk if the relationship dissolves during a partner's incarceration.
