# Supplementary material for: Decreasing HIV transmissions to African American women through interventions for men living with HIV post-incarceration: An agent-based modeling study
Source: PLoS One. 2019 Jul 15;14(7):e0219361. doi: 10.1371/journal.pone.0219361 (PMC6629075; doi:10.1371/journal.pone.0219361)
Supplement: S9 Table — (PDF) [file pone.0219361.s009.pdf]

S9 Table. Parameters varied within sensitivity analyses.

Variable	Estimate		
	Lower Bound	*Upper Bound*	*Base*
Duration of male high-risk behavior post-incarceration (months)	3	24	12
Assortative Mixing	0%	50%	30%
Duration of model run	10 years	n/a	20 years
Increase in sexual partners during high-risk period	None	n/a	See S6 Table
